# Secondary Metabolite Production Potential of Mangrove-Derived *Streptomyces olivaceus*

**DOI:** 10.3390/md19060332

**Published:** 2021-06-08

**Authors:** Dini Hu, Simon Ming-Yuen Lee, Kai Li, Kai Meng Mok

**Affiliations:** 1 School of Ecology and Nature Conservation, Beijing Forestry University, Beijing 100083, China; hudini@bjfu.edu.cn; 2 Department of Civil and Environmental Engineering, Faculty of Science and Technology, University of Macau, Macao, China; 3 State Key Laboratory of Quality Research in Chinese Medicine and Institute of Chinese Medical Sciences, University of Macau, Macao, China; simonlee@um.edu.mo

**Keywords:** mangroves, *Actinobacteria*, *Streptomyces*, whole genome sequencing, mass spectrometry, validamycin

## Abstract

Mangroves are intertidal extreme environments with rich microbial communities. *Actinobacteria* are well known for producing antibiotics. The search for biosynthetic potential of *Actinobacteria* from mangrove environments could provide more possibilities for useful secondary metabolites. In this study, whole genome sequencing and MS/MS analysis were used to explore the secondary metabolite production potential of one actinobacterial strain of *Streptomyces olivaceus* sp., isolated from a mangrove in Macau, China. The results showed that a total of 105 gene clusters were found in the genome of *S. olivaceus* sp., and 53 known secondary metabolites, including bioactive compounds, peptides, and other products, were predicted by genome mining. There were 28 secondary metabolites classified as antibiotics, which were not previously known from *S. olivaceus.* ISP medium 2 was then used to ferment the *S. olivaceus* sp. to determine which predicted secondary metabolite could be truly produced. The chemical analysis revealed that ectoine, melanin, and the antibiotic of validamycin A could be observed in the fermentation broth. This was the first observation that these three compounds can be produced by a strain of *S. olivaceus*. Therefore, it can be concluded that *Actinobacteria* isolated from the mangrove environment have unknown potential to produce bioactive secondary metabolites.

## 1. Introduction

Antibiotics are secondary metabolites with anti-pathogens and other beneficial activities; they are produced by microbes [1]. These substances can inhibit the growth of pathogenic microbes, thereby achieving the purpose of treating and preventing the various infections caused by them [2,3]. Antibiotics are mainly derived from *Actinobacteria*, among which, the largest genus, *Streptomyces*, produces 80% of them [1,4]. *Actinobacteria* have the potential to produce a great number of active substances. The unusual habitats where these bacteria live may have enabled them to develop unique metabolic systems and great secondary metabolite-producing capacities over their long evolutionary histories [5,6,7,8,9].

As extreme intertidal environments, mangroves are characterized by periodic tidal flooding, as well as strong winds and strong ultraviolet radiation. Much research attention has been directed at *Actinobacteria* from mangroves. Our research group has previously studied endophytic *Actinobacteria* from mangrove plants and was the first to report that *Streptomyces parvulus* can produce melanin and desferrioxamine B [10], a novel *Mycobacterium* species can produce asukamycin and apramycin [11], and *Micromonospora aurantiaca* can produce kanamycin [12]. In this study, we report on our continued investigation of potential secondary metabolite production by *Actinobacteria* from this special habitat. We selected a strain of *S. olivaceus* from the mangrove in Macau for whole genome sequencing to see if we could identify biosynthetic gene clusters and corresponding biosynthetic compounds in it. The fermentation products of this species were then analyzed by mass spectrometry to determine which predicted compounds it actually produced. Our results expand current scientific understanding of the secondary metabolite production ability of *Actinobacteria*.

## 2. Results and Discussion

Extreme ecological environments (e.g., mangroves) are considered natural reservoirs of abundant microbial resources with high biotechnological potential [13,14,15,16]. Alkaloid, macrolide, sesquiterpenoid, benzopyran, and cyclopentenone are the five main types of natural products isolated from mangroves. The first three are the most abundant and can serve as scaffolds for antibacterial drugs such as antibiotics [17].

### 2.1. General Genome Features

**Figure 1:**
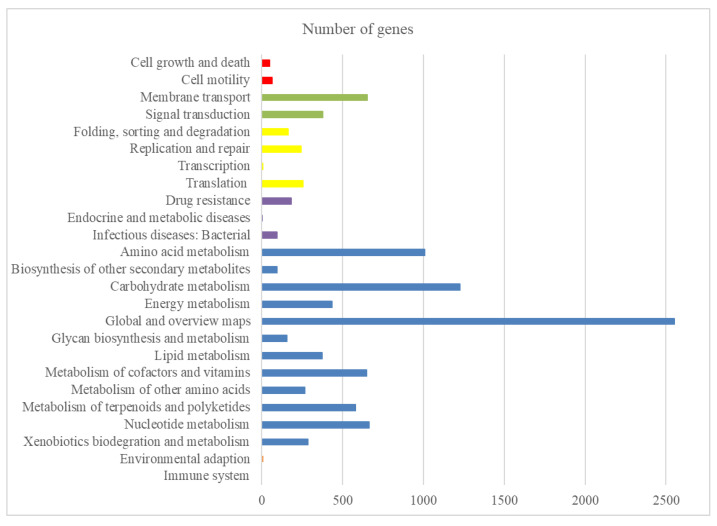
Distribution of KEGG pathways in the *S. olivaceus* sp. genome. The distribution of the predicted functional classification proteins was assigned by comparison with the KEGG database. Figure displays the five top KEGG Orthology (KO) categories of the assigned sequences, including organismal systems (orange), metabolism (blue), human diseases (purple), genetic information processing (yellow), environmental information processing (green), and cellular process (red).

The complete genome sequence from the *S. olivaceus* strain produced 7,172,349 reads and 291 scaffolds. De novo assembly was performed on these reads to generate a consensus sequence of 8,282,035 bp, with an average size of 28,461 bp and a G + C content of 72.38%. A total of 6968 protein-coding genes were found conserved in the genome. Altogether, 72 tRNAs were predicted, with an average coding sequence length of 1006 bp, and a coding density of ~87.77%. The Kyoto Encyclopedia of Genes and Genomes (KEGG) pathway analysis on the predicted proteins revealed that the top three categories in the functional classification were “global and overview maps”, “carbohydrate metabolism”, and “amino acid metabolism” (Figure 1).

### 2.2. Biosynthetic Gene Cluster Prediction

Nucleotide sequence analysis revealed that 105 gene clusters relating to the biosynthesis of 53 known secondary metabolites were present in the *S. olivaceus* strain’s genome (Supplementary Table S1). These biosynthesis cluster categories were predicted to be associated with the following: polyketides (PKS), non-ribosomal peptides (NRPS), amglyccycl, bacteriocin, ectoine, fatty acids, indoles, lantipeptide, melanin, nucleoside phenazine, saccharides, siderophores, terpenes, and other putative products. The predicted gene clusters from the *S. olivaceus* strain were significantly higher in number than those found in laboratory *Streptomyces* strains. For example, biosynthesis gene clusters from *S. coelicolor* A3(2) and *S. avermitilis* MA-4680, two model *Streptomyces* species, have over 20 gene clusters [18] and 30 gene clusters [19], respectively. Another *Streptomyces* species (*S. griseus* IFO 13350) has 34 secondary metabolite biosynthesis gene clusters [20]. In contrast, a similar number to those from the *Actinobacteria* isolated from mangroves. Moreover, 109 biosynthetic gene clusters were found in the *S. parvulus* strain [10], 105 gene clusters in a novel *Mycobacterium* strain [11], and 77 gene clusters in an *M. aurantiaca* strain [12]. The mangrove origins of the aforementioned bacteria have possibly heightened their secondary metabolite production abilities, making these organisms more adept at this function than their terrestrial counterparts. 

Within the *S. olivaceus* strain’s genome, 34 of the annotated gene clusters were associated with the production of 28 antibiotics (Supplementary Table S1). It is worth noting that four antibiotics produced by *S. olivaceus* (granaticin, elloramycin, and tetroazolemycins A and B) were not found in the genome sequence of our mangrove-isolated *S. olivaceus* strain. We also observed six predicted antibiotic compounds derived from non-*Streptomyces* genera, including *Bacillus*, *Micromonospora*, *Nonomuraea*, *Verrucosispora*, and an ‘uncultured bacterium’ (Supplementary Table S1). Therefore, our *S. olivaceus* strain from the mangroves in Macau not only had an increased ability (number of clusters) to produce secondary metabolites, but the characteristics of these compounds are expected to differ from the species and strains obtained elsewhere, too. The results suggest that the gene products from *Actinobacteria* isolated from the mangrove environment can participate in more diverse biosynthetic pathways than non-mangrove *Actinobacteria*. Thus, mangroves may be an ideal sampling site for isolating *Actinobacteria* with a greater productive range of bioactive compounds. 

### 2.3. Secondary Metabolites Produced by the S. olivaceus Strain

The *S. olivaceus* strain’s genome sequences raises the possibility of *S. olivaceus* spp. having the enormous potential to produce certain secondary metabolite types. However, to move beyond this conjecture, the predicted compounds were experimentally verified using MS/MS, which we developed to identify the secondary metabolites produced by the presence of the *S. olivaceus* strain grown in fermentation broth. The molecular ion peak of a compound can be obtained through a full MS/MS scan. However, the complex composition of the fermentation products necessitated us using a secondary mass spectrometer scan to detect the fragments and compare them with those in the published literature or in databases for final confirmation. We previously adopted this method to detect and analyze the secondary metabolites produced by three actinobacterial strains [10,11,12].

The mass spectral data from the positive mode was used to classify the metabolites in the fermentation broth, which were identified by the mass-to-charge ratio (*m/z*) of each molecular ion. The *m/z* values of the 53 predicted secondary metabolites were theoretically estimated with ChemDraw based on the structure in ChemSpider (http://www.chemspider.com accessed on 8 June 2021) (Supplementary Table S2). Because the chemical structures of seven of the compounds are not known (informatipeptin, phosphonoglycans, SAL-2242, SBI 06990 alpha/SBI 06989 beta, SCO-2138, spore pigment and SSV-2083), their theoretical *m/z* values could not to be estimated.

The profiles of the secondary metabolites present in the target strain were identified according to their theoretical *m/z* ratios because the theoretical values were similar to each other. The molecular ions of six of the predicted compounds were detected in the first scan mode (Supplementary Table S2). However, only ectoine, melanin, and validamycin (validamycin A), together with their corresponding in-source fragments, were observed in the second scan mode. Ectoine, a compound with an *m/z* ratio of 143.2, was found in the first scan mode (Supplementary Figure S1). In addition, the predicted reference fragment ions at *m/z* 68.2 and 97.0 was found in the second mode (Supplementary Figure S2), which matched with the results published by Fenizia et al. (2020) [21] (Supplementary Figure S3). Melanin, a compound with an *m/z* ratio of 319.3, was found in the first scan mode (Supplementary Figure S4), and fragment ions at *m/z* 301.2, 291.0, 289.1 273.2, 263.2, and 261.3 were found in the second scan mode (Supplementary Figure S5), which matched the reference *m/z* values predicted in the MS/MS spectrum (20V, positive) of melanin from the HMDB (Supplementary Figure S6). Furthermore, validamycin A, a compound with an *m/z* of 498.4, was found in the first scan mode, and fragments at 480.5, 466.2, 376.9, 318.5, 302.6, 299.6, and 276.1 *m/z* were found in the second scan (Supplementary Figures S7 and S8), which matched the reference *m/z* values predicted in the MS/MS of validamycin A from the HMDB (20V, positive) (Supplementary Figure S9). Thus, the production of ectoine, melanin, and validamycin A by the *S. olivaceus* strain was confirmed.

In our previous study, we used high-resolution mass spectra to confirm the accuracy of the 4000 Q TRAP MS/MS system [11]. In that study, two predicted secondary metabolites (asukamycin C and apramycin) were detected in the fermentation products. The theoretical m/z values of asukamycin C and apramycin were 521.22878 and 540.28809, respectively. The detected m/z values obtained by 4000 Q TRAP for asukamycin C and apramycin were 521.5 and 540.4, respectively, and they, therefore, had a ca. 0.2 difference in their m/z values like in the present study. Hence, a high-resolution LTQ-Orbitrap mass spectrometer with mass accuracy lower than 3 ppm was used to confirm the results. The measured mass values using the high-resolution mass spectrometer were 521.25092 and 540.22180 for asukamycin C and apramycin, respectively. Hence, the accuracy of 4000 Q TRAP system is sufficient for the purposes of this study.

By referring to previous studies, we discovered that this is the first time that these three compounds were produced by *S. olivaceus*. *S. chrysomallus* was the first species that was observed to produce ectoine, but industrial production of it depends on a strain of *Halomonas elongata* [22]. However, using *H. elongata* for ectoine production is far from straightforward, requiring rigorous experimental conditions and sophisticated equipment [22]. Melanins are generally black or brown pigments and are frequently used in pharmacology, medicine, and cosmetics preparations [23]. The melanin compound identified in the present study is consistent with that reported in a previous study by Hu et al. (2018) [10], who suggested that *S. parvulus* isolated from mangroves can produce melanin. Validamycin A, an aminoglycoside antibiotic used to control plant sheath blight caused by *Rhizoctonia solani*, was first isolated from *S. hygroscopicus* by Iwasa et al. in 1970 [24]. Interestingly, it was reported recently that validamycin A can effectively control Fusarium head blight in wheat [25,26], tomato Fusarium wilt, late blight, powdery mildew and cabbage black rot in field trials [27,28]. Bian et al. (2020) have unraveled the mechanisms of action for validamycin A, which occur through activation of the signaling pathways of Ca^2+^, reactive oxygen species, salicylic acid, jasmonic acid/ethylene, abscisic acid, and auxin in the plant [29]. Thus, the *S. olivaceus* strain obtained from mangroves herein is a potential alternative strain for producing the aforementioned compounds. In terms of identifying new microbial species that produce bioactive compounds, we conclude that the mangrove environment contains *Actinobacteria* that are able to produce bioactive secondary metabolites. Such metabolites may have uses in biotechnology (e.g., antibiotic production).

## 3. Materials and Methods

### 3.1. Isolation of the S. olivaceus Strain

The strain was isolated from *Kandelia candel* leaves from a mangrove environment in Macau. Our 16S rRNA sequence analysis showed that it shared 99.25% similarity with *S. olivaceus* NRRL B-3009^T^ [12]. The isolated *S. olivaceus* strain was stored in the State Key Laboratory of Quality Research in Chinese Medicine, University of Macau.

### 3.2. DNA Extraction and Whole Genome Sequencing

The DNA extraction, whole genome sequencing, and genomic analysis procedures followed those reported in a previous study [10]. Genomic DNA was prepared using the TIANamp Bacteria DNA Kit (TIANGEN Biotech Co. Ltd, Beijing, China). The genomic DNA library was constructed using the NEBNext Ultra II DNA Library Prep Kit for Illumina sequencing. The library was sequenced on the Illumina NovaSeq HiSeq 4000 instrument. Genome assembly and gene prediction were performed by IBDA and MetaGeneMark, respectively. tRNAscan-SE was developed for transfer RNA (tRNA) prediction. After searching against the KEGG database, functional categories were assigned throughout the genome. antiSMASH software was used to predict the biosynthetic gene clusters responsible for secondary metabolite production.

### 3.3. Fermentation Culture and Mass Spectrometric Analysis

The procedures used for the fermentation cultures and biochemical screening followed those described in a previous study [10]. The stored strain was transferred to ISP2 solid medium (containing, 4.0 g/L yeast extract, 10.0 g/L malt extract, 4.0 g/L dextrose, 20.0 g/L agar, pH 7.2) and cultured at 28 ℃ for 4 days. A single colony was inoculated into 20 mL of ISP2 liquid medium and the medium was cultured at 250 rpm for 7 days at 28 ℃. The fermentation broth were centrifuged (4000 rpm, 5 min) and the deposited cells were discarded. Crude extracts were prepared from the cultures by adding 60 mL of ethyl acetate to the fermentation products, and fractions of the resultant extracts were dried at 60 ℃, dissolved in 3 mL methanol, and then used for biochemical analysis. The concentration of the extract was 1000 ppm.

The 4000 Q TRAP tandem mass spectrometry (MS/MS) system (SCIEX, MA, U.S.A) used herein was equipped with a microflow electrospray (ESI) ionization mass spectrometer (MS) for separation and analysis of the extracellular secondary metabolites produced by the tested strain. Full scanning of the MS data was performed (m/z ratio, 100 to 1000; acquisition rate, 0.6 s per spectrum). The ESI source was operated in the positive mode (3.0 kV, capillary voltage; 20 V, cone voltage). A secondary mass spectrometric scan was performed for fragment detection. Metabolic profiling was compared with that in the Human Metabolome Database (HMDB, www.hmdb.ca accessed on 8 June 2021).

## 4. Conclusions

In this study, the biosynthetic potential of an *S. olivaceus* strain isolated from a mangrove environment was successfully investigated using a combination of whole genome sequencing and MS/MS analysis. This is the first report on ectoine, melanin, and validamycin A production in *S. olivaceus*. Thus, our results augment current scientific knowledge about endophytic *Actinobacteria* from mangroves, and provide a scientific basis for the full exploitation and utilization of secondary metabolites from endophytic *Actinobacteria* which can then be used for application in antibiotic production.

## Data Availability

The genome sequence of *S. olivaceus* sp. was deposited into the NCBI database under BioProject ID PRJNA677824.

## Supplementary Materials

The following are available online at https://www.mdpi.com/article/10.3390/md19060332/s1, Figure S1: The ethyl acetate extract of ectoine from the fermentation broth of *S. olivaceus* sp. were analyzed by MS/MS. Ectoine at [M+H]^+^ at *m/z* 143.2 were identified. The structure of ectoine was cross-referenced with the HMDB database, Figure S2: The ectoine ([M+H]^+^ at *m/z* 143.3), in-source fragments at *m/z* 68.2 and 97.0 were identified. The structures of the fragments were cross-referenced with Fenizia et al. (2020) [21] and drawn by ChemDraw., Figure S3: The fragments of ectoine identified in Fenizia et al. (2020) [21], Figure S4: The ethyl acetate extract of melanin from the fermentation broth of *S. olivaceus* sp. were analyzed by MS/MS. Melanin at [M+H]^+^ at *m/z* 319.3 were identified. The structure of melanin was cross-referenced with the HMDB database, Figure S5: The melanin ([M+H]^+^ at *m/z* 319.3), in-source fragments at *m/z* 301.2, 291.0, 289.1, 273.2, 263.2 and 261.3 were identified. The structures of the fragments were cross-referenced with the HMDB database, Figure S6: The fragments of melanin in HMDB database, Figure S7: The ethyl acetate extract of validamycin A from the fermentation broth of *S. olivaceus* sp. were analyzed by MS/MS. Validamycin A at [M+H]^+^ at *m/z* 498.4 were identified. The structure of validamycin A was cross-referenced with the HMDB database, Figure S8: The validamycin A ([M+H]^+^ at *m/z* 498.4), in-source fragments at *m/z* 480.5, 466.2, 376.9, 318.5, 302.6, 299.6 and 276.1 were identified. The structures of the fragments were cross-referenced with the HMDB database, Figure S9: The fragments of validamycin A in HMDB database, Table S1: The predicted biosynthetic gene clusters and corresponding secondary metabolites of *S. olivaceus* sp., Table S2: Predicted and detected values of *m/z* for the predicted secondary metabolites produced by *S. olivaceus* sp.
