# Supplementary figures and images for: Secondary Metabolite Production Potential of Mangrove-Derived Streptomyces olivaceus
Source: Mar Drugs. 2021 Jun 8;19(6):332. doi: 10.3390/md19060332 (PMC8228178; doi:10.3390/md19060332)

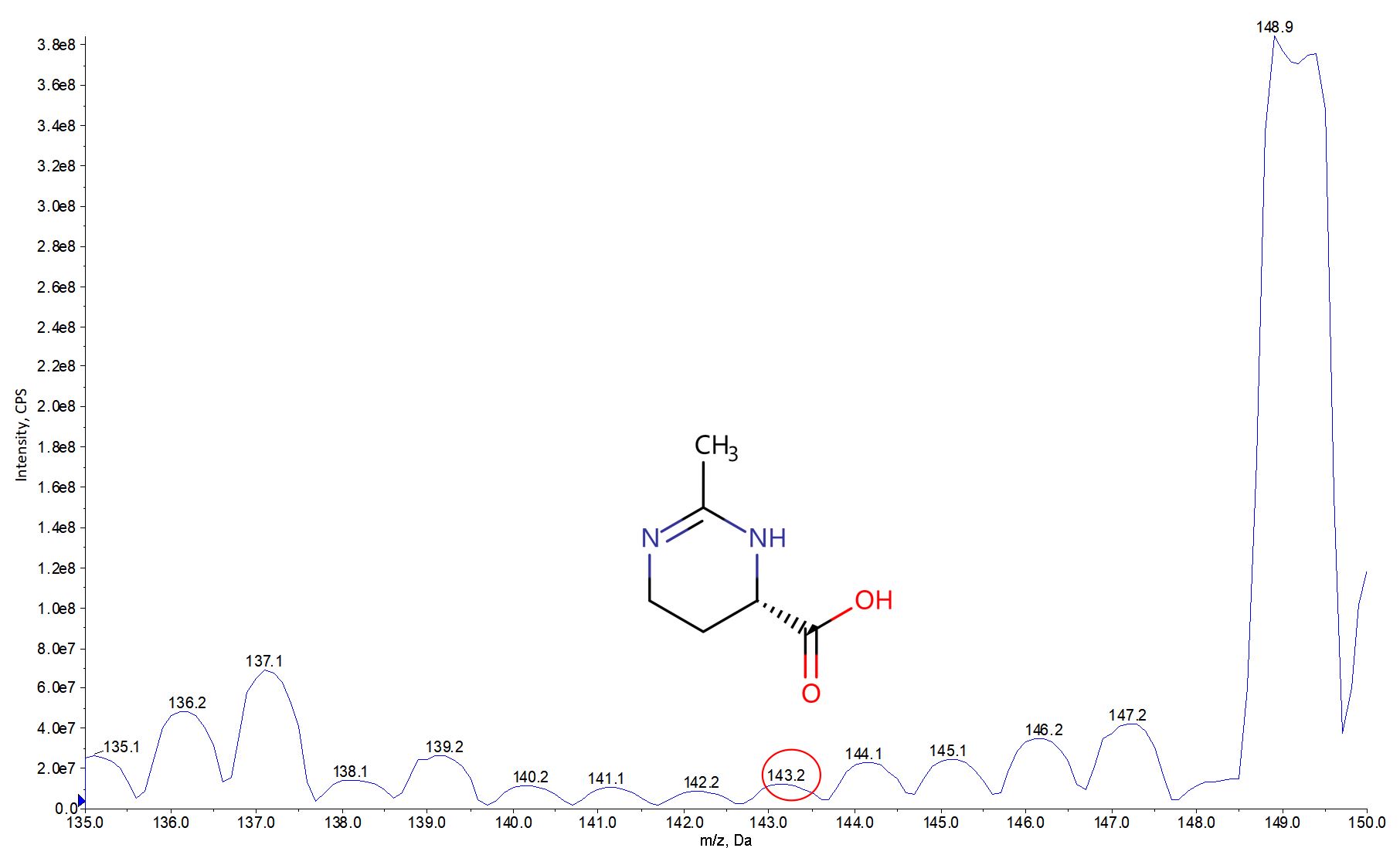

Supplement: Supplementary file 1 [file marinedrugs-19-00332-s001.zip › Supplementary Figure S1.JPG]

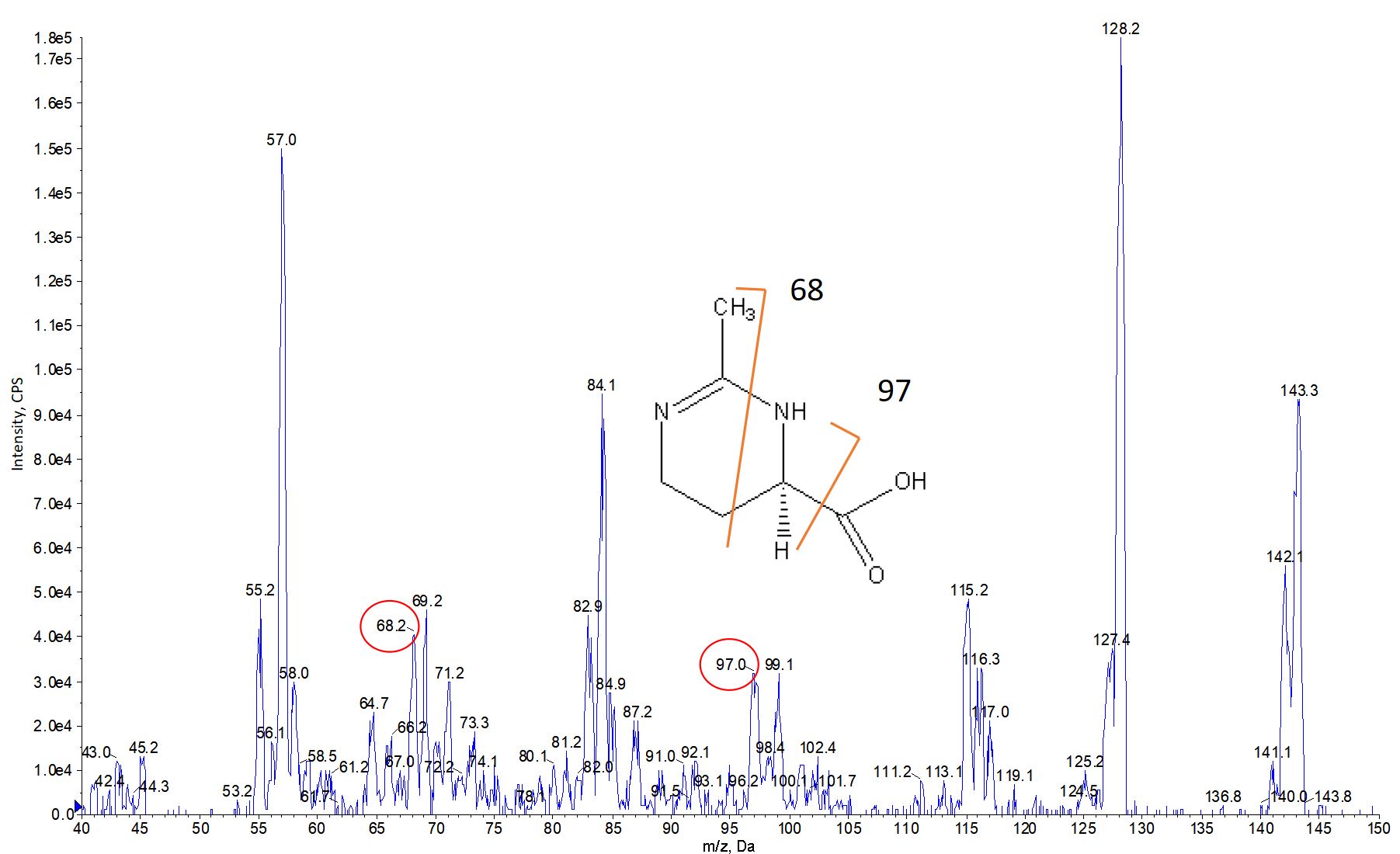

Supplement: Supplementary file 1 [file marinedrugs-19-00332-s001.zip › Supplementary Figure S2.JPG]

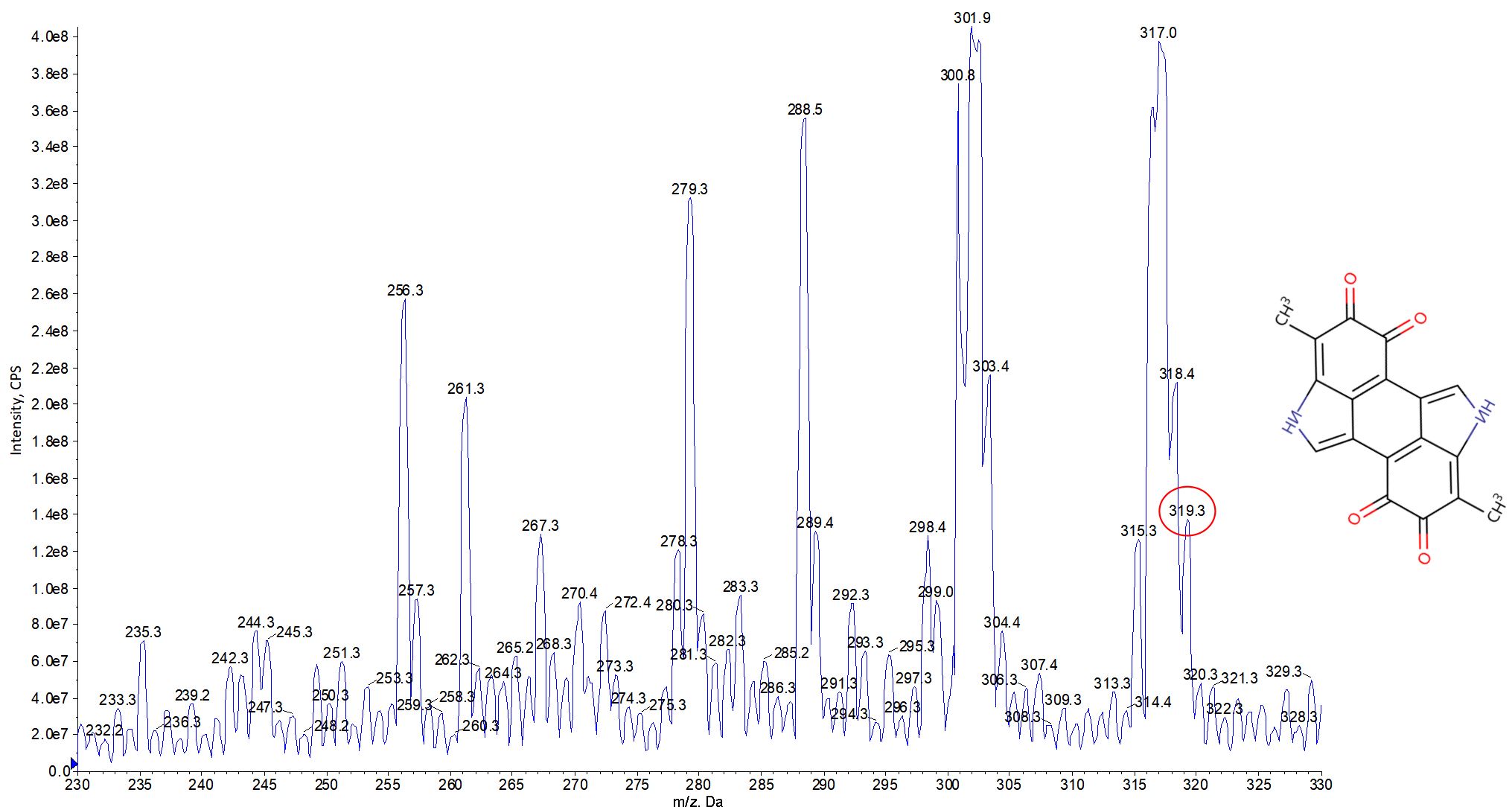

Supplement: Supplementary file 1 [file marinedrugs-19-00332-s001.zip › Supplementary Figure S4.JPG]

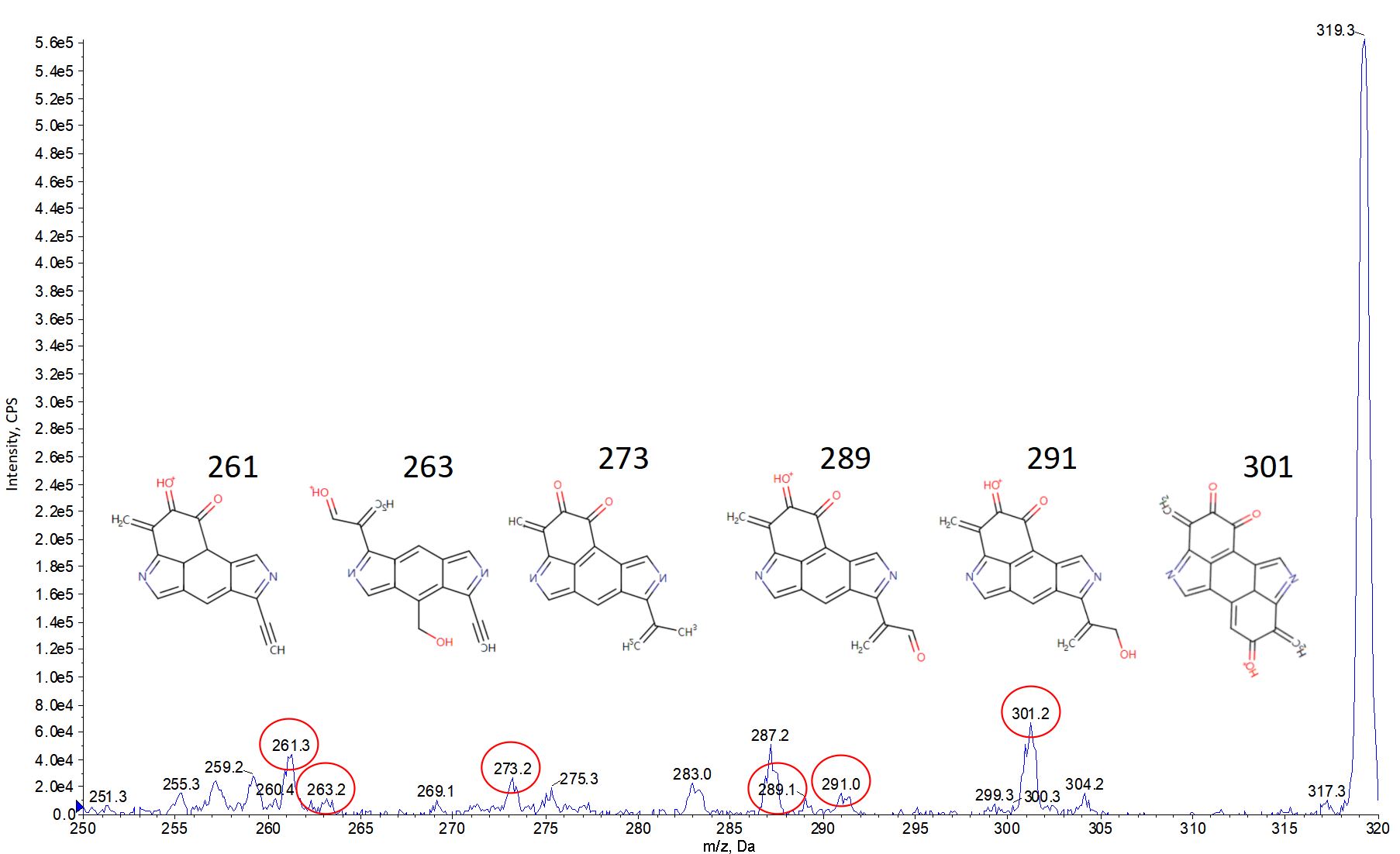

Supplement: Supplementary file 1 [file marinedrugs-19-00332-s001.zip › Supplementary Figure S5.JPG]

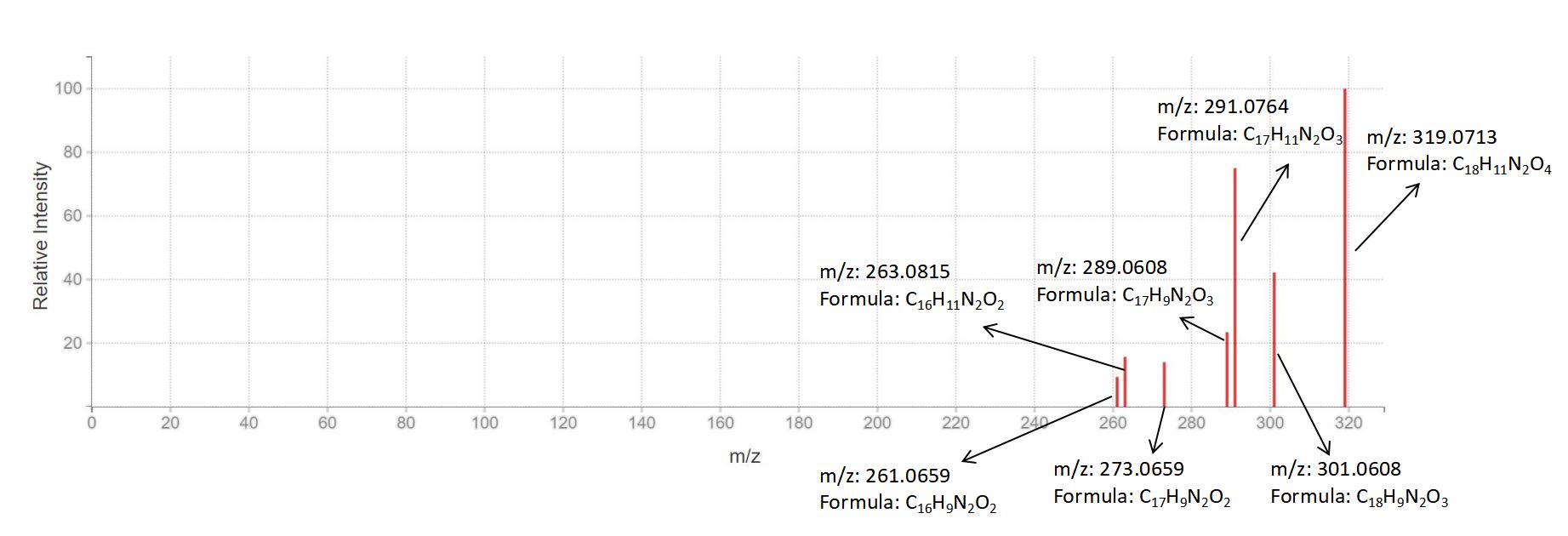

Supplement: Supplementary file 1 [file marinedrugs-19-00332-s001.zip › Supplementary Figure S6.JPG]

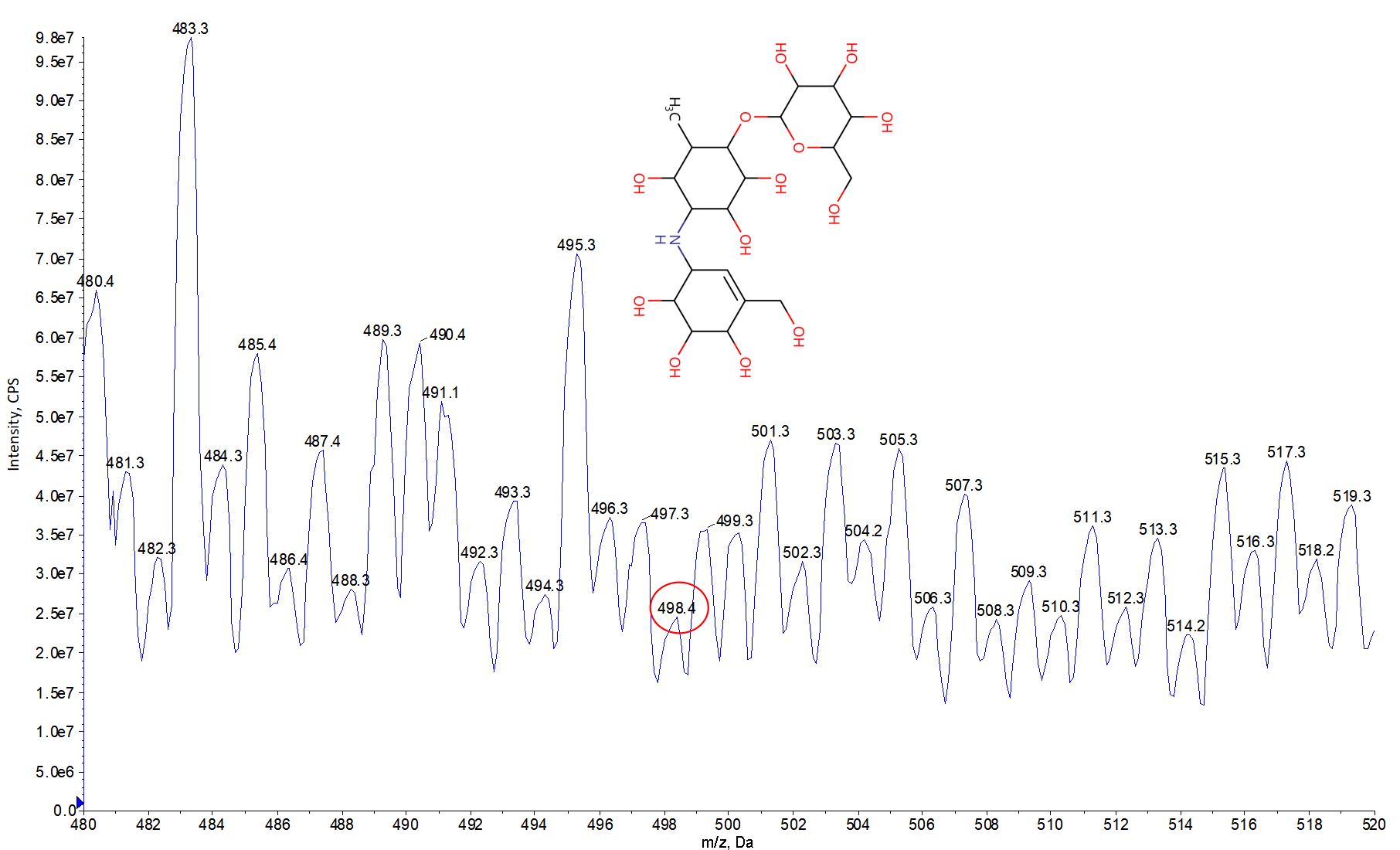

Supplement: Supplementary file 1 [file marinedrugs-19-00332-s001.zip › Supplementary Figure S7.JPG]

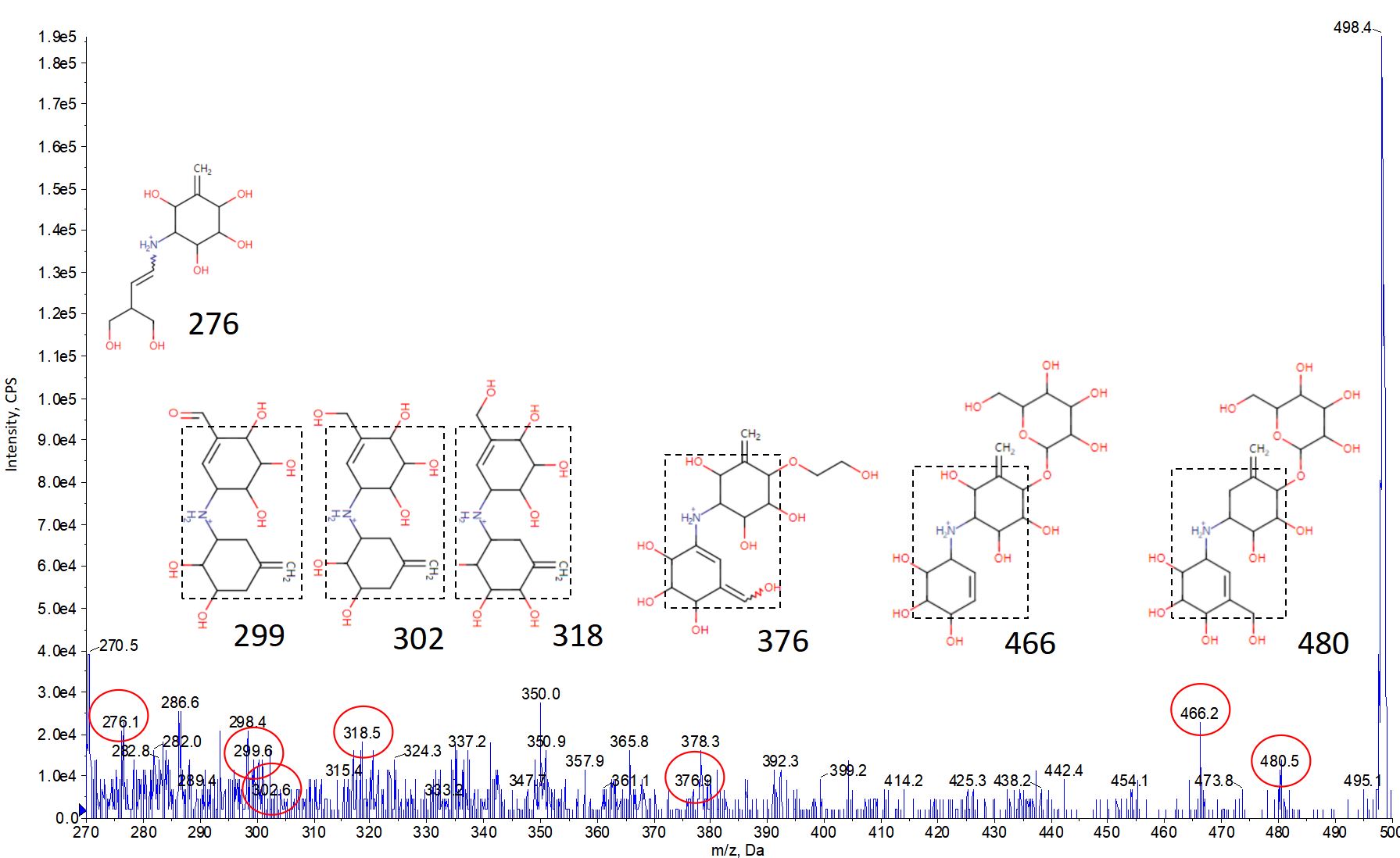

Supplement: Supplementary file 1 [file marinedrugs-19-00332-s001.zip › Supplementary Figure S8.JPG]

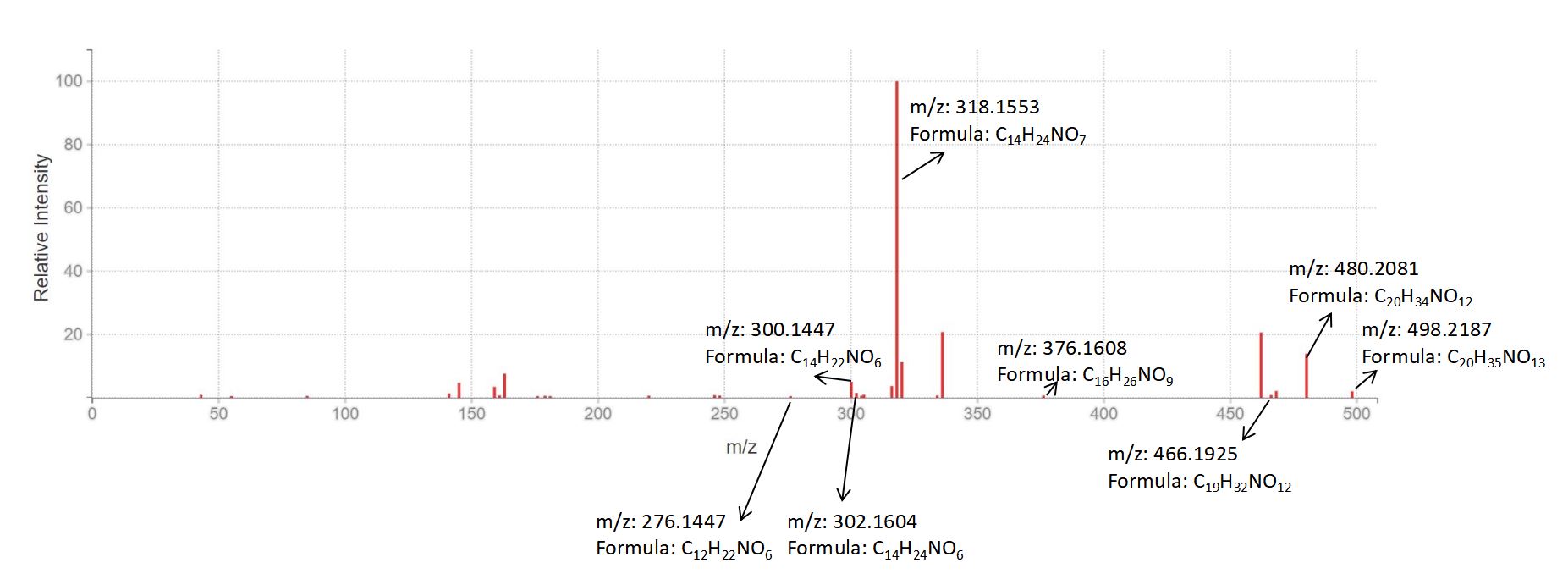

Supplement: Supplementary file 1 [file marinedrugs-19-00332-s001.zip › Supplementary Figure S9.JPG]

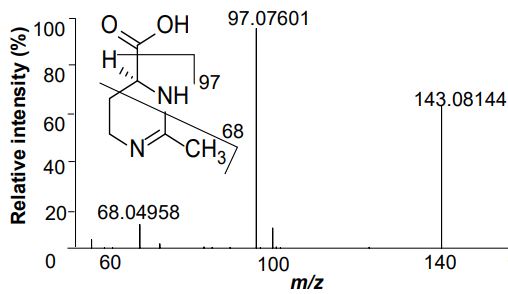

Supplement: Supplementary file 1 [file marinedrugs-19-00332-s001.zip › Supplementay Figure S3.JPG]
